# A Cooperative Intrusion Detection System for the Internet of Things Using Convolutional Neural Networks and Black Hole Optimization

**DOI:** 10.3390/s24154766

**Published:** 2024-07-23

**Authors:** Peiyu Li, Hui Wang, Guo Tian, Zhihui Fan

**Affiliations:** 1 Network and Informatization Office, Henan University of Science and Technology, Luoyang 471023, China; wh@haust.edu.cn (H.W.); tianguo@haust.edu.cn (G.T.); fzh@haust.edu.cn (Z.F.); 2 Henan Engineering Laboratory of Cloud Computing Data Center Network Key Technologies, Luoyang 471023, China

**Keywords:** cooperative intrusion detection system (CIDS), internet of things (IoT), convolutional neural network (CNN), black hole optimization (BHO)

## Abstract

Maintaining security in communication networks has long been a major concern. This issue has become increasingly crucial due to the emergence of new communication architectures like the Internet of Things (IoT) and the advancement and complexity of infiltration techniques. For usage in networks based on the Internet of Things, previous intrusion detection systems (IDSs), which often use a centralized design to identify threats, are now ineffective. For the resolution of these issues, this study presents a novel and cooperative approach to IoT intrusion detection that may be useful in resolving certain current security issues. The suggested approach chooses the most important attributes that best describe the communication between objects by using Black Hole Optimization (BHO). Additionally, a novel method for describing the network’s matrix-based communication properties is put forward. The inputs of the suggested intrusion detection model consist of these two feature sets. The suggested technique splits the network into a number of subnets using the software-defined network (SDN). Monitoring of each subnet is done by a controller node, which uses a parallel combination of convolutional neural networks (PCNN) to determine the presence of security threats in the traffic passing through its subnet. The proposed method also uses the majority voting approach for the cooperation of controller nodes in order to more accurately detect attacks. The findings demonstrate that, in comparison to the prior approaches, the suggested cooperative strategy can detect assaults in the NSLKDD and NSW-NB15 datasets with an accuracy of 99.89 and 97.72 percent, respectively. This is a minimum 0.6 percent improvement.

## 1. Introduction

The Internet of Things (IoT) is becoming one of the major communication-related concerns of today. In order to create a proper foundation for the rapid growth of the IoT in the real world, various academics have recently worked to tackle the obstacles in this sector due to the many benefits and uses of the IoT [1]. Every item in the IoT architecture has an identity and is capable of independent computing and communication [2]. One of the biggest issues with the delicate IoT applications in this scenario is security because, in real-world situations like smart cities [3], smart health networks [4], or industrial IoT [5], an attacker’s successful breach of this network and control over an object’s functionality might inflict irreversible harm.

IoT security is a sensitive topic that has drawn the attention of several researchers. However, recent research indicates that the solutions that have previously been given fall short of the ideal IoT security system [6]. However, this issue has become more complex due to the quick development of intrusion techniques and the emergence of fresh attack types [7]. The Intrusion Detection System (IDS) is regarded as one of the most effective security solutions for computer networks, since it often uses pattern analysis to identify attacks. Many IDSs are now available for IoT due to their ease of implementation and integration with IoT. To identify threats, however, a majority of the earlier models made use of centralized systems. In this instance, the Security Operation Centre (SOC) used the intrusion detection model [8].

These topologies may make the network more vulnerable in addition to raising costs and communication overheads (in case of failure or disconnection in the SOC). However, as compared to centralized models, the majority of earlier decentralized techniques had worse detection accuracy [9]. The goal of the present study is to address the aforementioned problems. By offering a cooperative IDS architecture, this study aims to lower the cost and overhead of communication in the intrusion detection process while simultaneously resolving the issues associated with IoT vulnerability. However, in contrast to centralized models, the suggested method’s usage of majority voting to identify infiltration has a guaranteed improvement in detection accuracy. This article’s contribution consists of the following:

- This article presents a feature selection technique based on Black Hole Optimization (BHO), whereby the smallest number of features required to represent an object’s communication is identified in a manner that minimizes the training error.
- The present study presents a matrix-based approach to characterize the network’s communication characteristics, which may improve the accuracy of attack detection.
- This study proposes a novel Parallel Convolutional Neural Network (PCNN)-based architecture for IoT local attack detection. Two CNNs make up the aforementioned model; the first CNN processes the features chosen by BHO, while the second CNN processes communication features in matrix form. To identify the presence of an attack, the characteristics from these two CNN models are combined and a SoftMax layer is added.
- The PCNN model is used locally by each intrusion detection agent in this article’s Cooperative Intrusion Detection (CID) architecture, which is built on Software Defined Networking (SDN) in the IoT. In this paradigm, to identify the presence of assaults, participating controllers in the communication between each pair of objects communicate their local detection findings and vote by majority.

This article’s continuation is structured as follows: Research records are reviewed in Section 2. The suggested CID solution using deep learning (DL) and optimization methods are shown in Section 3. Section 4 delves into the discussion of the outcomes of the proposed method, and Section 5 presents the conclusion.

## 2. Research Background

This section provides an analysis of the literature on IDSs for IoT. However, before going through specific works, it is necessary to note that the question of security metrics, which would allow one to assess the efficiency of an IDS, is crucial. Some of the previous research have attempted to establish the relationship between quantifiable attributes and the security of IoT devices. Kuk et al., 2020, used object-oriented software metrics to detect the presence of code vulnerability. According to their findings, certain metric values may correspond to possible security vulnerabilities [10]. Setzler and Mountrouidou, 2021, proposed a more extensive approach, and thus they created new security metrics based on some features of IoT devices [11]. Their work stresses the need for automation in the measurement of security in testbeds as a way of getting repeatable metrics.

An IoT device named BIoTIDS that used blockchain technology was introduced in [12]. This technology was capable of identifying distributed denial of service assaults on the Internet of Things in addition to identifying the attacker. A set of criteria derived from user transaction patterns in the blockchain network can identify attackers in this system.

A suggested IDS for the IoT that used CNN and fuzzy logic was presented in [13]. Using this strategy, the collection of attributes associated with assaults was initially selected using the information gain (IG) criteria. The feature selection procedure was then finished by applying fuzzy rules to the features with high IG. Ultimately, the fuzzy model’s output was combined with the collection of chosen attributes to identify the CNN assault. There have been a lot of IDSs made available for IoT in recent years. While deep learning (DL) approaches have garnered significant interest for intrusion detection, machine learning (ML) techniques have been used for detection in the bulk of these systems.

An IDS in IoT, based on cloud computing and machine learning, was provided by the study done in [14]. With this approach, an effort was made to concentrate on cutting down on cloud computing expenses and the learning model’s learning time. For this reason, a lightweight intrusion detection model known as MFE–ELM was proposed, integrating the techniques of Multi-Feature Extraction (MFE) and an Extreme Learning Machine (ELM). An ELM that is fed via many MFE characteristics is called the MFE–ELM model. While this approach can outperform conventional learning models like artificial neural networks (ANN) and support vector machines (SVM), the accuracy numbers provided here do not demonstrate any improvement over previous techniques.

A DL-based IDS for heterogeneous IoT networks was presented in [15]. In this study, pre-processed features were given to a CNN learning model in vector form. Two one-dimensional convolution layers made up this CNN model, and a max pooling layer was positioned after each one. Lastly, a SoftMax layer classified the feature maps that this CNN extracted. When it comes to identifying Distributed Denial of Service (DDoS) assaults, this strategy performs well.

In [16], the Chaotic Cuckoo Search Optimization Algorithm (CCSOA) was used to choose characteristics related to presence of attacks in IoT. Based on the amount of chosen features and the training error criterion, this optimization method selected features. After that, assaults were identified using a classification model based on the Optimal Wavelet Kernel Extreme Learning Machine (OWKELM). The Sunflower Optimization Algorithm (SOA) was used by the OWKELM model that was presented in this study in order to optimize the parameters.

The use of DL approaches to identify IoT threats was examined in [17]. Using binary coding, this approach first normalized the input characteristics before converting them into a matrix format that was suitable with deep models. The effectiveness of three deep models—deep neural network (DNN), long short-term memory (LSTM), and CNN—in identifying IoT assaults was then compared. The study’s findings demonstrated that, in comparison to the other two models, the CNN model can identify assaults more successfully and correctly.

To protect IoT against threats, the IDS described in [18] combined blockchain technology with DL approaches. The effectiveness of the IDS could be increased by combining blockchain with machine learning, but the model’s use was unknown due to its complexity and the need to set it up on the blockchain network.

In [19], infiltration in IoT was detected by combining CNN and Capuchin Search Algorithm (CapSA). This approach extracted data feature maps by using a CNN model after pre-processing the input features. The collected feature vectors’ dimensions were further decreased using CapSA. The training error and the amount of chosen features in this feature selection technique were referred to as optimization targets. The random forest (RF) was used to categorize the characteristics and identify the attack once the feature had been chosen.

A Modified Isolated Forest (M-iForest)-based method for identifying abnormalities in IoT networks was proposed in [20]. This approach used two different components to identify anomalies after preprocessing the input characteristics. The M-iForest learning approach helped both elements; however, the second component was based on normal samples, while the first component was based entirely on attack samples. Then, using the compliance of the combined patterns, the results of the detection of these two components were combined to accomplish anomaly detection. DL methods were used in [21] to detect the encroachment of IoT-based smart agriculture applications. Recursive feature elimination was the initial step in this method’s reduction of the input features. After that, color pictures were created using the remaining set of characteristics. Lastly, feature categorization and attack detection were performed using the three VGG16, Inception, and XReption models. The VGG16 model performed better than the other two models, according to the findings that have been published.

Researchers in [22] put forward a federated Network IDS (FN-IDS) which applied federated learning to solve the privacy issue in the distributed network traffic data environment. It was also advantageous where data privacy was an issue of concern since data were not being transmitted over the internet. However, the performance of FN-IDS might be somehow restricted by the type of machine learning algorithms used for attack classification.

Researchers in [23] presented an extensive literature review of ML and DL in IoT-IDS in a more extensive study. This work categorized various intrusions and also discussed various approaches of implementing and monitoring IDSs in the IoT infrastructure. This was useful in getting an overall view of the market of security solutions for IoT.

Authors in [24] proposed a new IDS for IoT networks using deep learning for anomaly-based intrusion detection with filter-based feature selection using DNN. This approach solved one of the major issues of anomaly detection, namely that, in most datasets, normal network traffic is orders of magnitude larger than attack data. Thus, by creating synthetic data for minority attack classes, they obtained high detection accuracy.

## 3. Methodology

Three processes are often seen in machine learning-based intrusion detection systems (IDSs), namely preprocessing, feature selection/extraction, and classification. Essentially the same pattern is used in this study’s recommended method for IoT intrusion detection. Nonetheless, the suggested method’s classification process is carried out by having the learning components share local findings. The computing processes of the suggested technique are then provided after the databases’ structures are explained. These databases are utilized to train the suggested learning model and identify assaults.

### 3.1. Database

Samples from two databases, NSLKDD [25] and NSW-NB15 [26], were utilized in this investigation. In the realm of intrusion detection, one of the most used databases is the NSLKDD data collection. The purpose of this database is to address the issues with KDD’99 data collecting. Each data record in this database has one non-dependent variable (attack type) and forty-one independent variables. Three characteristics in this collection are nominal—Service, Flag, and ProtocolType; the other attributes are numerical. The number of samples in the NSLKDD database was 1,074,992. After the many duplicate entries in this collection were eliminated, a data set with over 126,000 records was produced. Twenty percent of the samples in this database were utilized in this investigation.

Along with regular traffic data, the NSW-NB15 database is another more recent intrusion detection data collection that covers nine different forms of assaults. There are 2,540,044 samples in this dataset. Each sample is described by 49 distinct characteristics, which are further classified into two categories, flow-based features and packet-based features, depending on their nature. The uneven samples of various classes in this database are one of its issues; over 87% of the samples are from the normal class. This concern was addressed in this research by using 20% of the samples. In the reduction procedure, the excluded samples were chosen from the normal class. After that, there were 508,000 records in the NSW-NB15 database, of which 321,283 samples were associated with different assaults and the remaining samples were associated with the normal class.

These two datasets have been widely used in IDS applications in the IoT. Even though both these datasets are concerned with more conventional network traffic and do not necessarily capture the subtleties of today’s IoT interactions, they provide generalized benefits. For example, features such as type of protocol, the services that have been requested, and the size of the packets, (while not limited to IoT devices) can be used to detect malicious activities and possible attack patterns that may be applicable to the compromised IoT devices. Also, these datasets offer well-defined standards for the identification of anomalies. These datasets, when used to train an IDS model, create a baseline of what constitutes normal network traffic, and this can be used to develop an IDS that can identify IoT-specific anomalies.

### 3.2. Proposed Cooperative Intrusion Detection System (CIDS)

A CIDS should take advantage of an efficient architecture for network deployment and, at the same time, use a powerful learning model to detect attacks. To meet these requirements in the proposed method, the network is divided, and the intrusion detection components are deployed using an SDN-based technique. Each detection component additionally makes use of the BHO combination and PCNN models. As a result, there are three primary stages to the suggested CIDS:

1. Formation of subnets based on SDN
2. Local detection based on BHO and PCNN
3. Intrusion detection based on Majority Voting

**Figure 1:**
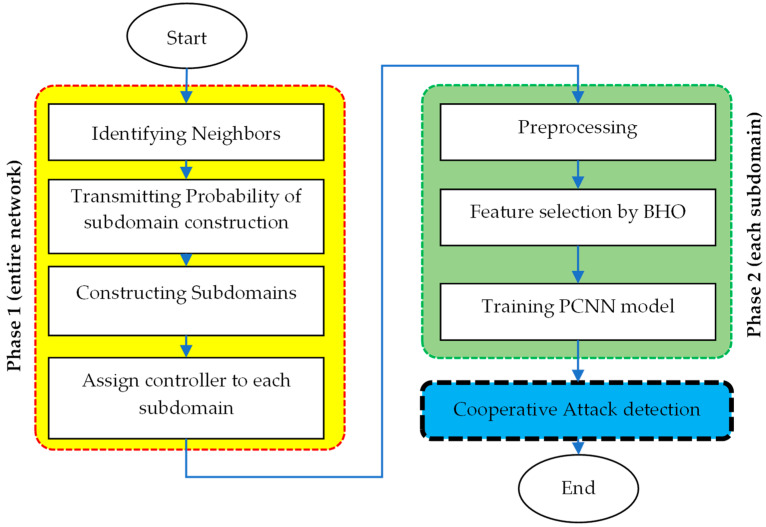
The steps of the proposed method.

Figure 1 shows the suggested technique’s diagram. The first stage of the suggested approach divides the network into a series of subnets according to object positions by applying it globally to the whole network. This phase is illustrated on the left side of the diagram in Figure 1 and starts by identifying neighbors of each active node in IoT. Then, the probability of constructing a subdomain is calculated by each node, after exchanging control packets with the identified neighbors. Based on the calculated probabilities, the network is divided into several subdomains. This phase is finalized by assigning a controller node to each subdomain in the network. The second phase, which is implemented locally in each subnet and illustrated on the right side of the diagram, analyzes shared traffic data across subnets to detect the presence of attacks. This phase starts by preprocessing traffic features and then selecting an optimal subset of features which is performed using BHO. The selected features are then used to prepare the training data for two CNNs which form a parallel architecture for intrusion detection. The resulting trained PCNN model is used by the subdomain’s controller for analyzing the traffic flows passing through it and identifying the presence of attacks. Ultimately, the third stage is executed by means of collaboration between the subnets that exchange data.

The proposed CIDS architecture exploits some features that make it suitable in protecting IoT communication networks as described below. First, it is possible to have distributed processing of tasks within the resource-scarce IoT devices using the SDN-based approach. Centralized IDS models from the traditional approach may not perform well with scaling issues and computational issues that arise with a large number of interconnected devices [27]. Since the network is divided into subnets, and there are controller nodes assigned for the CIDS, the load is distributed well and is suitable for IoT use.

Secondly, the CIDS uses BHO for feature selection. This is important because IoT network traffic may have different characteristics compared to conventional network interactions. Due to BHO’s capability of extracting the relevant features from the data stream, the CIDS can effectively focus on the patterns that are likely to be indicative of potential attacks on the IoT vulnerabilities.

Last of all, the ability to classify using PCNN further strengthens the CIDS’ suitability for IoT. PCNNs can make good use of both the vector characteristics and the matrix characteristics existing in IoT communication data, so it has a stronger attack detection ability than simple models. The remainder of this section goes over each stage of the proposed approach.

#### 3.2.1. Formation of Subnets Based on SDN

We investigated an Internet of Things network with many mobility heterogeneous nodes arranged according to SDN. A central SDN controller and numerous distributed controllers are part of the presumptive SDN architecture. One-hop or multi-hop connections are used to link the controller nodes to the central controller. It is assumed that every controller node has a fixed location and stable connectivity with every other controller node. IoT nodes are separated into a number of subnets in the first stage of the suggested technique in order to provide a framework for the intrusion detection elements. Finding each object’s neighbors is the first step in the network division process. To do this, every object broadcasts a HELLO control packet to its neighbors, announcing its existence to them. Following receipt of this message, every object replies to the transmitter node with its ID. Thereafter, a list of all network objects’ neighbors is created. Any object having at least one active neighbor generates a random number, say R, in the interval [0, 1], and multicasts it to its neighbors via a control packet, say RVP, once the deadline for receiving the Hello control packet has passed. Every node in the network receives many RVP packets carrying the R values of its neighbors by means of this procedure being repeated by all of the active objects in the network. Every node starts the process of constructing a subdomain if its R-value is greater than the values it receives from its neighbors after comparing its own R-value to those values. In this manner, the node uses an SDC control packet to multicast its energy data and the count of its active neighbors to its neighboring nodes. Each node that creates the subnet in this way has the potential to receive one or more SDC packets. The node joins the subnet associated with the transmitter object if it only receives one SDC packet. However, in the event that the node has received several SDC packets, it uses the following relation to order the options:

(1) $$Score\left(i\right)=\frac{E_{SDC_{i}}}{\sum_{j=1}^{\left|SDC\right|}E_{SDC_{j}}}+\frac{N_{SDC_{i}}}{\sum_{j=1}^{\left|SDC\right|}N_{SDC_{j}}}$$

where |SDC| indicates the total number of messages received, and $E_{SDC_{i}}$ and $N_{SDC_{i}}$ stand for the energy information and the number of neighbours taken from the i-th SDC message. The node with the highest computed score, determined by using Equation (1), will be included in the subnet after the computation of the scores for each potential selection. Following the aforementioned process, the network splits up into many subnets. Every subnet node communicates the associated nodes’ information to the closest controller node, which forwards this structure to the central controller. The subnet formation procedure is repeated on a regular basis and if one of the established structure’s connections is broken. It should be mentioned that in the created structure, the controller, which is in charge of overseeing the subnet, handles all data exchange. To transfer data to node B, for example, an object like A must first provide communication information with node B to the controller overseeing its subnet. The data packet is then delivered to the controller overseeing node B’s subnet via communication between the controller nodes. It is feasible to effectively monitor all network traffic thanks to this routing procedure.

#### 3.2.2. Local Detection Based on BHO and PCNN

The learning components found in the controller nodes are used to identify assaults locally in the second step of the proposed strategy, which follows network deconstruction. The following is the procedure by which controllers keeping an eye on every subnet identify local attacks:

- Pre-processing
- Feature selection based on BHO
- Feature description and local detection based on PCNN

The input data are transformed into a format that may be processed for the next stages of the suggested procedure during the pre-processing stage. The BHO method is used in the second stage to identify the ideal subset of characteristics. In order to reduce the training error, this method seeks to choose the fewest features feasible. In the third phase, each input record in the suggested PCNN model is represented as a vector or matrix that serves as the input for a parallel CNN. Local attack detection is done based on these factors.

##### Preprocessing

Data preparation is the first stage in each subnet’s local attack detection procedure. The suggested approach pre-processes data records by transforming nominal values into numerical values. In order to do this, a list of the distinct values for each nominal feature is created, and the items in this list are arranged in ascending order according to the quantity of repetitions in the training data set. Next, a natural number that corresponds to each element’s position in the sorted list is used to replace it. All data records are now characterized as numerical vectors as a consequence of this procedure. Additionally, zero is substituted for any missing values in the feature if there are any in the data.

##### Feature Selection Based on BHO

The BHO algorithm selects features in the second stage of the local assault detection phase. Black holes and stars’ motion served as inspiration for the BHO algorithm. Every potential solution in this method is represented as a star, and the local optimum takes the shape of a black hole. The main characteristics of this optimization technique are its efficient search of the problem space and the absence of the necessity for many parameters, because the BHO method keeps candidate solutions from becoming stuck at local optima by simulating how the black holes behave when they eat surrounding stars. The suggested technique selects or eliminates features based on each solution vector in the BHO algorithm. The selection mode of each feature is thus regarded as an optimization variable in order to treat the feature selection issue as an optimization problem. Stated differently, every solution vector is represented as a binary vector with a length equal to the number of independent features (49 for NSW-NB15 and 41 for NSLKDD) in the database. Assigning a zero to a feature in an optimization variable of the solution vector indicates its elimination, whereas assigning a one indicates its selection. Two subsets of 10% of database samples were utilized as training and validation data to assess each solution vector’s fitness. To do this, the training data are first used to extract the set of features chosen for the solution vector, and then the learning model is trained using those features. Next, using the validation samples as a foundation, the trained model’s error is computed. In the suggested strategy, the BHO algorithm’s goal is to identify the smallest subset of characteristics that may be used to minimize the learning model’s error. Accordingly, depending on the error criterion and the quantity of features, the fitness function in the suggested feature selection method may be defined as follows:

(2) $$fitness(x)=a\times \frac{F}{N}+(1-a)\times \frac{\sum_{i=1}^{K}x_{i}}{K}$$

where K is the number of features and x is the input solution vector. Additionally, N denotes the quantity of validation samples, and F represents the quantity of validation samples for which an incorrect attack prediction was produced. In the end, parameter a represents the significance coefficient of the error criterion and the quantity of features; in the suggested approach, its value is fixed at 0.8. The following are the feature selection phases that the BHO algorithm in the suggested technique does in accordance with the structure that is provided for the solution vectors and how to assess their fitness:

Step 1: Generate a random initial population of solution vectors in the form of binary strings.

Step 2: Calculate the fitness of each solution vector based on Equation (2).

Step 3: Determining the solution with the least fitness as a black hole $X_{BH}$.

Step 4: Move the location of each solution such as $X_{i}$ as [28]:

(3) $$X_{i}=X_{i}+rand.(X_{BH}-X_{i})$$

The black hole’s position in (3) is indicated by vector *X_BH_*, while the star/solution *i*’s location in the problem space is shown by the vector *X_i_*. A random number in the interval 0–1 is also represented as rand.

Step 5: Determine [28] the star’s threshold distance to be engulfed by the black hole.

(4) $$R=\frac{fitness(BH)}{\sum_{i=1}^{N}fitness(i)}$$

where *N* is the BHO algorithm’s total number of solution vectors.

Step 6: Replace a solution vector with the current black hole if its fitness is lower than that of the black hole.

Step 7: Substitute a new random solution vector for any solution vectors whose distance from the black hole is less than the threshold *R*.

Step 8: Proceed to the next step if the algorithm’s iteration count is beyond *T*; if not, resume your search from Step 2.

Step 9: As the best-found answer, return the black hole with the lowest fitness.

Following the aforementioned procedures, the subset that defines the ideal characteristics is thought to be the black hole with the lowest fitness. The characteristics designated as features picked by the BHO algorithm are those indicated with a number one in the optimum solution for this purpose.

##### Feature Description and Local Detection Based on PCNN

**Figure 2:**
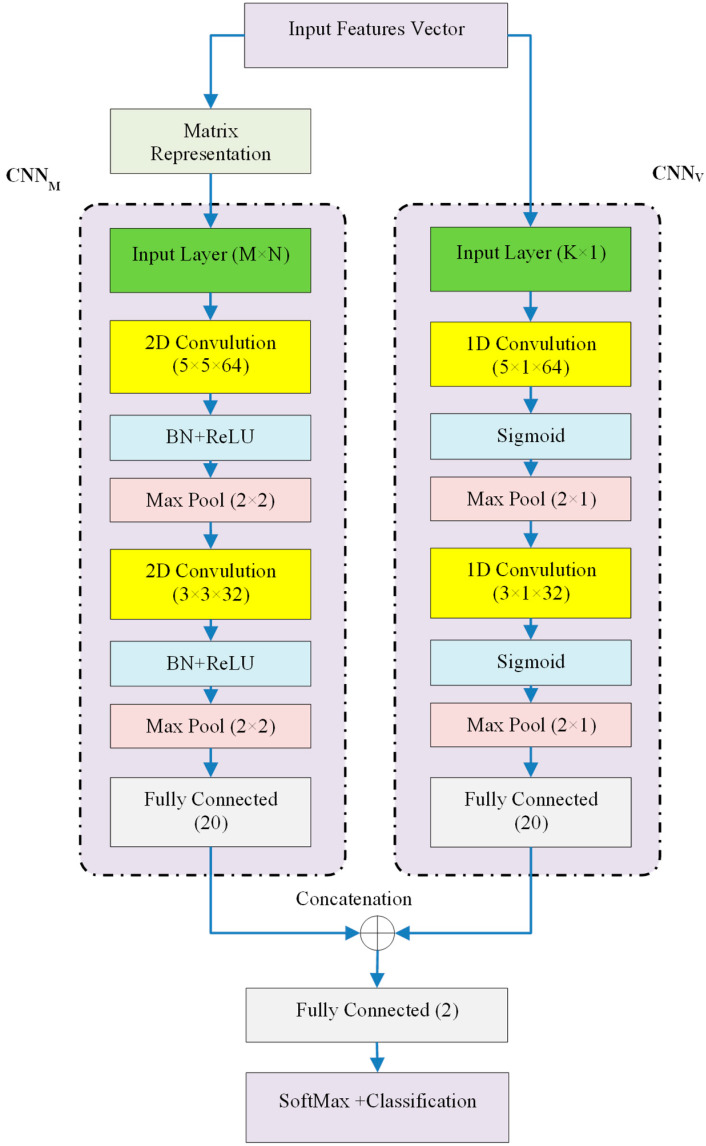
Proposed PCNN model for local attack detection in controller nodes.

Every controller node employs a PCNN model in the third stage of the local attack detection phase to identify assaults. The two parallel CNN models that make up the suggested PCNN model are separately utilized to extract features from vector and matrix data types. Figure 2 depicts the suggested PCNN model’s structure.

The suggested PCNN model, as shown in Figure 2, is composed of two parallel models, CNNV and CNNM, which process the input features’ vector and matrix representations, respectively. Two convolution layers, followed by activation and pooling layers, comprise both CNN models. Lastly, fully connected layers are used to transform the feature maps that CNNV and CNNM retrieved into vectors with a length of 20. These two vectors are concatenated and then classified using a SoftMax layer in the following. The CNNV model’s convolution layers are one-dimensional since it takes the pre-processed features in vector form, unaltered. Moreover, activation functions such as sigmoid functions are used. The CNNM model, on the other hand, receives its input characteristics as a matrix representation. This is why the model’s activation functions are identified as ReLU type and its convolution layers are two-dimensional. Prior to supplying input for each ReLU layer, a Batch Normalisation (BN) layer is also used. It should be emphasized that the layers of these two models were configured experimentally, with the configuration that produced the best training performance being employed. The matrix form of the input characteristics used to feed the CNNM model is explained below. The following relationship is used to normalize the values of each pre-processed chosen feature before the input feature vector is transformed into a matrix form:

(5) $$\bar{\vec{X}}=\frac{\vec{x}-x_{min}}{x_{max}-x_{min}}$$

where the normalized vector is described by $\bar{\vec{X}}$ and the input feature vector is represented by $\vec{x}$. Additionally, the feature vector $\vec{x}$’s lowest and maximum values are represented, respectively, by $x_{min}$ and $x_{max}$. Each feature’s values are transferred to the [0, 1] range once each feature has been normalized. This interval is split into 20 ranges of length 0.05 in the following, such as [0, 0.05), [0.05, 0.1), [0.95, 1], and the one-hot coding technique is used to each feature value to create a binary string of length 20. Here, every value in the interval [0, 0.05] is transformed into a binary string where the first bit is always one. Additionally, only the second bit in the binary string representing the values in the range [0.05, 0.1] is equal to one, with the other 19 bits being zero. In the end, every value in the interval [0.95, 1] is transformed into a binary string, of which the last bit is always one. Every database value is processed in this manner, resulting in a 20-bit description for every attribute. This means that 820 bits are used to describe each NSLKDD database record, and 980 bits are used to describe each NSW-NB15 database record. Subsequently, each record’s derived bit strings are separated into groups of eight, converting each set of eight bits into an image pixel. Twelve zero bits are added to the end of each bit string of each NSLKDD database record, and twenty-eight zero bits are added to the end of each NSW-NB15 database record since the bit strings of each record are not multiples of eight. This gives each NSLKDD database record a description in the form of a 13 × 8 matrix, representing a picture of 104 pixels. In contrast, the picture that is produced when every NSW-NB15 database entry is represented has 126 pixels, or a 14 × 9 matrix. Based on the suggested PCNN model, the generated matrices are fed into the CNNM model to carry out local attack detection.

#### 3.2.3. Intrusion Detection Based on Majority Voting

**Figure 3:**
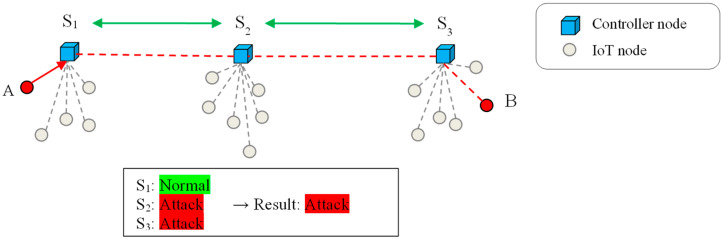
CID process in the proposed method based on a hypothetical scenario.

The suggested method’s third step involves using majority voting to identify intrusions. Every time there is a traffic flow between network nodes, this step is carried out. In order to do this, every controller node engaged in delivering a traffic flow conducts local attack detection using the PCNN model and thereafter transmits the results of its detection to the other controllers involved. The presence of an assault is then ascertained by means of a majority vote among the outputs that are derived from the controller nodes. In this scenario, the controller nodes will terminate the connections whose voting outcome results in an attack. Figure 3 illustrates the CID in the suggested approach using a fictitious situation.

Figure 3 considers a traffic flow produced by three imagined controller nodes, S1, S2, and S3, between two objects, A and B. These three nodes communicate their local detection results to identify intrusion based on the suggested strategy. Based on the majority vote, this flow is categorized as an attack, and the link between A and B is cancelled. This figure shows that the detection results of controllers S1, S2, and S3 were “normal”, “attack”, and “attack” respectively.

## 4. Results and Discussions

To put the suggested approach into practice, MATLAB 2020a was used. Throughout the studies, a 500 m × 500 m network of 400 heterogeneous nodes was postulated, where each node’s radio range, buffer capacity, mobility speed, and beginning energy were distinct from one another. Fifty percent of the nodes were mobile, and their starting positions were determined at random. The nodes’ starting energy was adjusted between 0.5 and 1 joule, and their movement speed was adjustable between 0.5 and 2 m per second. For every database, the simulation procedure was carried out ten times. PCNN models were trained using 90% of the database samples in each iteration, with the remaining 10% being utilized to assess how well the models detected novel assaults. In this instance, one of the test records was used to identify the communication characteristics between each pair of nodes in the simulation process. It should be mentioned that the effectiveness of the suggested model was evaluated using fresh samples for each iteration. In this manner, all database samples were utilized for model testing after ten rounds. It should be mentioned that the BHO algorithm’s feature selection settings in both databases were set to 300 iterations and 100 population size, respectively, throughout the trials.

Precision, accuracy, recall, and f-measure criteria were used to assess the effectiveness of the IDS. Following the simulation, the labels assigned to the test samples by the classification model were compared to the real labels of those samples. The accuracy criteria indicate how well the system detects both normal and attack-related samples simultaneously by displaying the ratio of successfully categorized samples to the total number of test samples. F-measure, recall, and precision, on the other hand, concentrate on accurately identifying assaults. Using the precision criterion, one can ascertain the proportion of accurate outputs identified as attacks by the IDS. Conversely, the recall criterion indicates the percentage of attacks that the IDS accurately identified. In conclusion, the f-measure criterion evaluates the system’s overall efficacy in attack detection by calculating the harmonic mean of two recall and precision criteria as follows [29]:

(6) $$Precision=\frac{TP}{TP+FP}$$

(7) $$Recall=\frac{TP}{TP+FN}$$

(8) $$F-measure=\frac{2\times Precision\times Recall}{Precision+Recall}$$

(9) $$Accuracy=\frac{TP+TN}{TP+TN+FP+FN}$$

Regarding the relationships stated above, TP denotes the number of attacks correctly detected by the IDS, and FN is the number of attacks that the IDS failed to detect. Additionally, FP represents the quantity of normal instances incorrectly categorized as attacks, and TN refers to the number of normal instances correctly identified by the IDS. IDS functionality should strive to optimize the aforementioned criteria. In order to demonstrate the efficacy of each technique employed in the proposed method, their performance was juxtaposed with the subsequent scenarios:

- Proposed (CNNv only): Local attack detection is limited to vector features and the proposed CNNv model for each controller node in this scenario. It is important to acknowledge that in the given situation, the collection of controller nodes continues to collectively identify the presence of an attack.
- Recommended (CNNM only): In this scenario, local attack detection is exclusively carried out by each controller node using the matrix features and CNNM model. Furthermore, the voting process among the controller nodes for the ultimate attack detection is solely determined by the output of the CNNM models.
- Proposed (non-cooperative): In this scenario, the proposed PCNN model is utilized by individual controller nodes to identify attacks within their respective subnets; the collaborative voting and intrusion detection processes are disregarded.

**Figure 4:**
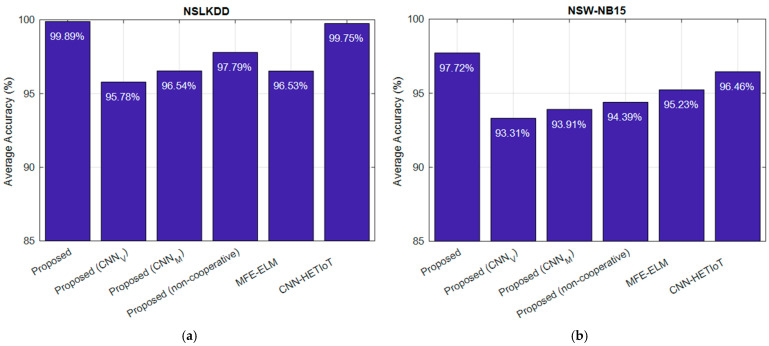
Comparison of attack detection accuracy in two databases (**a**) NSLKDD and (**b**) NSW-NB15 by the proposed method with other methods. MFE-ELM [14], CNN-HETIoT [15].

Furthermore, a comparative analysis was conducted between the outcomes of the proposed method and those of the MFE–ELM [14] and CNN–HETIoT [15] approaches. The accuracy of attack detection in the NSLKDD and NSW-NB15 databases using the proposed method was compared to that of alternative methods, as illustrated in Figure 4.

The results presented in the diagrams of Figure 4 can be utilized to assess the efficacy of each technique implemented in the proposed method. The presented graphs illustrate that the proposed approach, which integrates PCNN and cooperative detection, achieves an average accuracy of 99.89% in classifying samples as either normal or attack. This is the case despite the fact that the CNNv and CNN_M_ models can independently classify the database samples with 95.78 and 96.54 percent accuracy, respectively.

As a result, it can be deduced that, to begin with, transforming the features into matrices may enhance the detection accuracy. This is due to the functional characteristics of convolutional neural network (CNN) models, which are particularly well-suited for the processing of image data. Furthermore, the integration of the two CNN models, as denoted by PCNN, results in a minimum 3.35 percent enhancement in accuracy. The amalgamation of the features extracted by these two Convolutional Neural Network (CNN) models may yield a more precise depiction of the features, thereby augmenting the detection accuracy.

**Figure 5:**
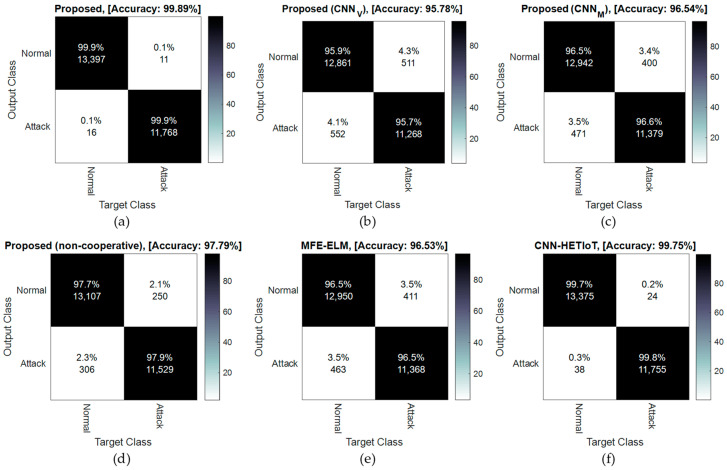
Confusion matrix resulting from attack detection in the NSLKDD database by the (**a**) proposed method, (**b**) CNN_V_, (**c**) CNN_M_, (**d**) the proposed method in non-cooperative mode, (**e**) MFE-ELM [14], and (**f**) CNN-HETIoT [15].

Conversely, the accuracy is diminished by 2.1% if the cooperative approach to detecting attacks is disregarded and the detection is carried out independently by each controller node. Consequently, it can be asserted that the voting methodology employed in the suggested CIDS has the potential to enhance the precision of network traffic categorization. Due to the fact that the collaboration among the controller nodes and majority voting among their outputs enables the utilization of alternative models to compensate for the local error introduced by each learning model, analyzing the values documented in the NSW-NB15 database produces comparable outcomes. The results indicate that the proposed cooperative solution in both the NSLKDD and NSW-NB15 databases can improve detection accuracy; this enhancement pertains to the methods utilized in the proposed method. The confusion matrices produced by the classification of NSLKDD database samples are illustrated in Figure 5. The columns of these matrices correspond to the legitimate identifiers, while the rows symbolize the outcomes of each algorithm’s classification. The proposed method misclassified a total of 27 samples out of 25,192, according to the confusion matrices derived from the classification of NSLKDD database samples. Of these, eleven were attacks that the cooperative model proposed by the method failed to detect, and sixteen were the outcome of false alarms generated by the proposed CIDS. The performance of the CNN–HETIoT method [15], which achieved a detection accuracy of 99.75%, is most comparable to that of the proposed method. A total of 62 error samples are generated by this procedure. In comparison to the closest method, the proposed method reduces the number of false alarms and undetected attacks by more than fifty percent, as demonstrated by these results.

**Figure 6:**
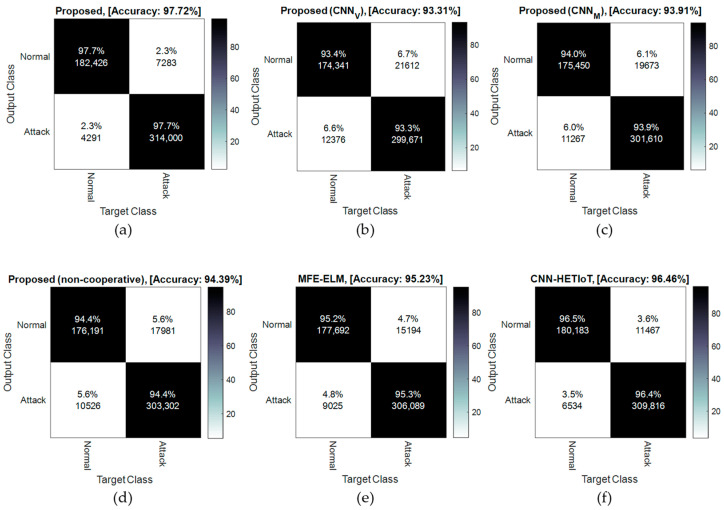
Confusion matrix resulting from attack detection in NSW-NB15 database by the (**a**) proposed method, (**b**) CNN_V_, (**c**) CNN_M_, (**d**) the proposed method in non-cooperative mode, (**e**) MFE-ELM [14], and (**f**) CNN-HETIoT [15].

The confusion matrix resulting from the detection of attacks in the NSW-NB15 database using various methodologies is depicted in Figure 6. When the classification results in Figure 5 and Figure 6 are compared, it is evident that NSLKDD outperforms all other methods in terms of accuracy when classifying NSW-NB15 samples. The potential cause for this could be ascribed to the increasingly intricate pattern of novel assaults documented in the NSW-NB15 database. However, in comparison to the alternative approaches, the proposed procedure exhibited a reduced degree of accuracy loss. This superiority can be attributed to the application of the following two effective techniques: the first is the proposed method’s utilization of a parallel combination of CNN models in order to generate a more comprehensive feature map. In this particular scenario, CNN models can enhance the accuracy of detection by collaborating on feature maps. As a result, the proposed model has achieved greater accuracy than the MFE–ELM model [14], even when operating in non-cooperative mode, for NSLKDD. Furthermore, it is comparable in performance to the aforementioned method for the NSW-NB15 dataset. Utilizing a cooperative detection technique based on consensus between learning agents situated in the controller nodes is the second benefit of the proposed method. Voting has been extensively implemented in ensemble learning models, and it has been demonstrated theoretically that this method can improve detection efficacy in comparison to the non-cooperative mode [30]. The experimental results provide confirmation of this notion. False alarms and missed detections of models with greater error can be reduced in the proposed method through majority voting between the outputs of learning components. As a result, the accuracy of the proposed method in the cooperative mode for the NSW-NB15 database is 3.33% greater than in the non-cooperative mode. Analogous to the NSLKDD database, the proposed method classifies normal and assault samples in the NSW-NB15 dataset. The rate of undetected assaults and false alarms using the proposed method is 2.3% (total error = 11,574 samples), as shown in Figure 6a. Conversely, the CNN-HEIoT method [15] exhibits a false alarm rate of 3.6% and an undetected attack rate of 3.6% (for a total error of 18,001 samples). This indicates that, relative to the closest method, the proposed technique can reduce classification errors by 35.7%.

**Figure 7:**
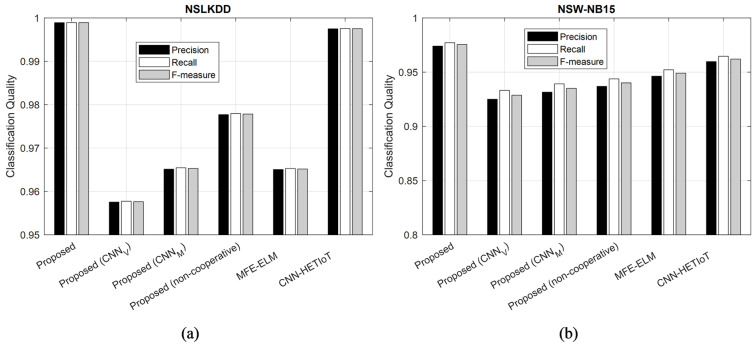
Evaluation results of different methods based on precision, recall, and F-Measure criteria for (**a**) NSLKDD and (**b**) NSW-NB15. MFE-ELM [14], CNN-HETIoT [15].

The outcomes of assessing various approaches according to the criteria of precision, recall, and f-measure are illustrated in Figure 7. The findings presented in this figure are the consequence of classifying samples from the NSLKDD and NSW-NB15 data sets. The presented figure illustrates how the suggested approach outperforms NSLKDD and NSW-NB15 in terms of f-measure, precision, and recall when applied to attack detection on both datasets.

The increased precision values of the proposed method indicate a greater likelihood that the positive outputs produced by this method are accurate. Thus, the likelihood of false alarms is reduced in the proposed method in comparison to the methods under consideration. Conversely, the increased recall criterion validates that the suggested approach successfully identified a greater frequency of network attacks. In accordance with the f-measure criterion, the performance of the proposed method is superior to that of the contrasted methods due to its recall and precision. By prioritizing the detection of assaults, the proposed method achieves a higher quality of classification, as demonstrated by these results.

**Figure 8:**
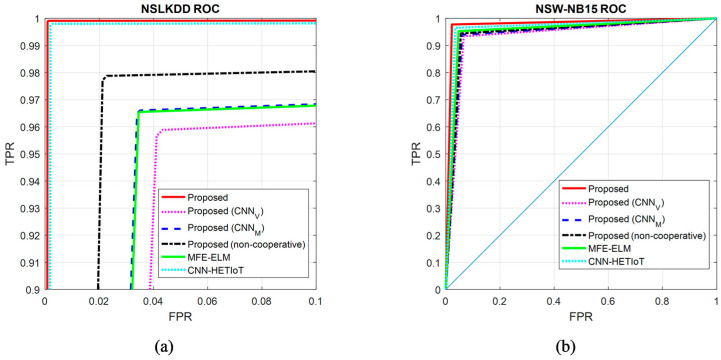
ROC curve resulting from the classification of (**a**) NSLKDD and (**b**) NSW-NB15 samples. MFE-ELM [14], CNN-HETIoT [15].

Figure 8 illustrates the ROC curve that was generated through the process of classifying the samples from the two databases utilized in this study. It is important to mention that the ROC curve, which is illustrated in Figure 8a, is magnified due to the classification of NSLKDD samples. The graphical representations depicted in this figure validate the conclusions derived from the research outcomes. The graphs illustrate that although the proposed method decreases the occurrence of false positives (or false alarms), it may result in a rise in the rate of true positives. The numerical values derived from the experiments conducted in this section are presented in Table 1.

The results of this study demonstrated that the cooperative system under consideration is an effective method for identifying breaches in IoT-architected networks and can be implemented to detect intrusions in real-world situations.

### Discussion, Limitations, and Future Works

IoT networks have been considered in this research, and a new CIDS was developed with the help of CNNs and BHO. The results of the implementation show that the discussed model can be used to protect IoT environments. This section presents the discussion on the major findings, limitations, and recommendations for future research.

A. Discussion

The results show that the proposed CIDS has a high accuracy rate; above 99% for NSLKDD and 97% for UNSW-NB15 in attack detection. This goes to show how an IDS can use BHO for feature selection and PCNN for classification as a good combination. The PCNN model uses both vector and matrix-based characteristics which enables the model to provide a more holistic view of the network traffic for attack detection. This architecture results in an accuracy enhancement of at least 3.35 percent, compared to the individual CNN models on both datasets. Also, the majority voting technique among the controller nodes in the CIDS is evidently proved to enhance the accuracy by at least 2.1% compared to the non-cooperative detection technique. This helps to reduce possible errors in individual nodes of the system thus improving its reliability of the intrusion detection system. Comparing the efficiency of the proposed model with hybrid approaches, such as MFE–ELM [14] and CNN–HETIoT [15], proved its superiority in more accurate identification. Although NSLKDD and UNSW-NB15 are good sources of data, they do not mimic current IoT communication to the fullest extent. However, the findings could demonstrate the capability of the introduced CIDS to adapt these features for attack detection in IoT scenarios.

B. Limitations and Future Works

Even considering the capabilities of the introduced CIDS, there are still the following areas to further improve to counter the existing problems of the IoT environment:

- Dataset Limitations: NSLKDD and UNSW-NB15 mainly rely on traditional network traffic and may not cover all the details of the current IoT protocols and devices’ communications.
- Scalability: Regarding the current implementation, it is important to observe that the approach is based on the static network size. Perhaps, when expanding the CIDS to accommodate large and evolving IoT networks the distributed processing and messaging paradigms may require further study.
- Computational Cost: Training PCNN models is computationally expensive, especially when dealing with big data. These may include a study on new lightweight CNN architectures or effective training methods for IoT devices.

As for the future work, there are several directions of research that can improve the CIDS for practical applications of IoT, including the following:

- The future work may involve using realistic IoT network traffic or other particular IoT datasets to enhance the identification of attacks on IoT vulnerabilities.
- Future works may include research on how other controllers can be trained using distributed learning for PCNN models. This can help overcome the problem of scalability and may, at the same time, help to decrease the time needed for training.
- Examining the federated learning techniques in which the controller nodes jointly update the CIDS model without transmitting raw data could be explored in future works. This can help in large-scale IoT applications in solving issues of privacy.

Such limitations and further research directions can be used to enhance the effectiveness and usability of the CIDS for the security of the IoT networks’ communication.

## 5. Conclusions

This article introduces a novel approach to cooperative attack detection within the Internet of Things (IoT). SDN is employed in the proposed approach to partition the network into a collection of subnets. Local attack detection in each subnet is executed by the learning model housed within its controller. To accomplish this, the most pertinent attack-related features are initially chosen using BHO. Subsequently, a PCNN model is implemented to ascertain whether an attack is present in every subnet. The proposed PCNN model is comprised of two CNN models that analyze traffic features in vector and matrix formats concurrently. The concatenation and classification of the feature set obtained from the two CNN models are performed by employing SoftMax and classification layers. Following the completion of local intrusion detection by the controller nodes, the ultimate detection result is determined through majority voting. Experiments conducted for this study indicate that the parallel combination utilized in the proposed CNN models may be capable of enhancing detection precision. In order for the proposed PCNN model to enhance accuracy by a minimum of 3.35% when compared to the scenario in which individual CNN models are employed in isolation for attack detection. Conversely, when compared to the centralized/local approach, the proposed cooperative approach and attack detection via voting among network controllers can enhance detection accuracy by a minimum of 2.1%. The findings of this study demonstrate that every technique utilized in this research, which sets it apart from prior investigations, has proven to be efficacious in enhancing the efficacy of the IDS. Using the databases, NSLKDD and NSW-NB15, to evaluate the performance of the proposed method revealed that it is feasible to detect attacks with an accuracy of 99.89 and 97.72 percent, respectively, which is at least a 0.6% improvement over previous research. Conversely, the evaluation of recall and precision metrics substantiates the efficacy of the suggested approach in identifying communications that result in malicious activities.

## Figures and Tables

**Table 1:** Comparing the efficiency of the proposed method with other methods.

Database	Method	Accuracy	F-Measure	Recall	Precision
NSLKDD	Proposed	99.8928%	0.9989	0.9989	0.9989
	Proposed (CNN_V_)	95.7804%	0.9576	0.9577	0.9575
	Proposed (CNN_M_)	96.5426%	0.9653	0.9655	0.9651
	Proposed (non-cooperative)	97.7930%	0.9778	0.978	0.9777
	MFE–ELM [14]	96.5306%	0.9652	0.9653	0.9651
	CNN–HETIoT [15]	99.7539%	0.9975	0.9976	0.9975
NSW-NB15	Proposed	97.7217%	0.9756	0.9772	0.9741
	Proposed (CNN_V_)	93.3094%	0.9288	0.9332	0.925
	Proposed (CNN_M_)	93.9094%	0.9351	0.9392	0.9316
	Proposed (non-cooperative)	94.3884%	0.9401	0.9438	0.9369
	MFE–ELM [14]	95.2325%	0.9491	0.9522	0.9463
	CNN–HETIoT [15]	96.4565%	0.9621	0.9647	0.9598

## Data Availability

Data are contained within the article.
